# Barrier Modification of Metal-contact on Silicon by Sub-2 nm Platinum Nanoparticles and Thin Dielectrics

**DOI:** 10.1038/srep25234

**Published:** 2016-04-28

**Authors:** Haisheng Zheng, Bikram K. Mahajan, Sheng C. Su, Somik Mukherjee, Keshab Gangopadhyay, Shubhra Gangopadhyay

**Affiliations:** 1 Department of Electrical and Computer Engineering, University of Missouri-Columbia, Missouri 65201, USA; 2 Nanos Technologies LLC, Columbia, Missouri 65203, USA.

## Abstract

We report metal/p-Si contact barrier modification through the introduction of either “isolated” or “nonisolated” tilted-target-sputtered sub-2 nm platinum nanoparticles (Pt NPs) in combination with either a 0.98 nm Atomic Layer Deposited Al_2_O_3_ or a 1.6 nm chemically grown SiO_2_ dielectric layer, or both. Here, we study the role of these Pt NP’s size dependent properties, i.e., the Pt NP-metal surface dipole, the Coulomb blockade and quantum confinement effect in determining the degree of Fermi level depinning observed at the studied metal/p-Si interfaces. By varying only the embedded Pt NP size and its areal density, the nature of the contact can also be modulated to be either Schottky or Ohmic upon utilizing the same gate metal. 0.74 nm Pt NPs with an areal density of 1.1 × 10^13^ cm^−2^ show ~382 times higher current densities compared to the control sample embedded with similarly sized Pt NPs with ~1.6 times lower areal densities. We further demonstrate that both Schottky (Ti/p-Si) and poor Ohmic (Au/p-Si) contact can be modulated into a good Ohmic contact with current density of 18.7 ± 0.6 A/cm^2^ and 10.4 ± 0.4 A/cm^2^, respectively, showing ~18 and ~30 times improvement. A perfect forward/reverse current ratio of 1.041 is achieved for these low doped p-Si samples.

Metal-semiconductor (M-S) interfaces are fundamental to semiconductor based devices and can deeply influence the device performance. Based on decades of research exploring the nature of the M-S interface, most metals form a rectifying Schottky contact on both n- and p-type semiconductors with a Schottky barrier height (SBH). This barrier height has been defined, as the energy mismatch between the majority carrier band edge and the Fermi level of the metal in contact^1^. Experimental evidence has revealed over the years that regardless of the metal work function, the barrier at the M-S interface tends to pin the Fermi level of the semiconductor to a particular value. The term “Fermi level pinning” was introduced to describe this insensitivity of the SBH to the metal work function. Many researchers have tried different approaches to explain the mechanism of Fermi level pinning. Some of the more prevalent of these approaches are the defect model, the metal induced gap states (MIGS) or interface trap states (ITS), chemical bonding model, or inhomogeneity of Schottky barriers, etc.^1^,^2^,^3^,^4^,^5^. As a result of Fermi level pinning, the contribution of contact resistance due to non-ideal Ohmic contact affects the device characteristics drastically in the nanoscale regime^6^. Among various methods to overcome this issue, doping the semiconductor heavily to reduce effective barrier width is the most common one. However, the doping methods used (e.g. ion implantation, diffusion) do not have a precise control of depth and anisotropy, and hence, cannot be reliably applied in the case of precise doping of organic semiconductors or 2D material based devices. Numerous studies have also demonstrated reduction of M-S contact resistance upon incorporation of a thin insulating tunnel barrier with fixed charges^7^,^8^. Incorporation of a thin dielectric layer between the M-S interface reduces the SBH by partially ‘depinning’ the Fermi level; but the additional dielectric barrier on the other hand reduces the tunnelling probability. Thus, the oxide thickness must be optimized to achieve a favourable trade-off between the SBH and tunnelling probability. However, the thin dielectric layer can only partially depin the Fermi level. The reported optimal insulating layer thickness (e.g. ~1 nm for Al_2_O_3_) is difficult to fabricate, yet thicker insulator layers increase the contact resistance by reducing the tunnelling probability. Dielectric layers below this thickness (~1 nm) are unreliable due to surface discontinuities^9^.

The concept of barrier modification using two metals with different SBH was introduced in the 1990s when Tung^10^ reported the first analytic solution of electron transport in inhomogeneous Schottky barrier heights (ISBH) with the prediction of the potential pinch-off effect. The curvature of the interface dipole along the direction in perpendicular to the contact (z direction), however, is ignored in ISBH model which may induce errors on the estimation of the field enhancement. In the 2000s, Narayanan^11^ introduced the notion of enhanced tunnelling at the triple interface (ETTI) due to the M-M interface interaction. These models have been adopted, theoretically and experimentally, by many researchers to explain the barrier modification due to metal nanoparticles (MNPs) at the MS contact^12^,^13^,^14^,^15^,^16^,^17^,^18^. Based on the ISBH and ETTI models, the interface dipole layer formed between the MNPs and the metal electrode (due to the difference in their work function) can enhance the localized electric field (E-field) resulting in the reduction of the SBH and current crowding effect. It can be predicted that reducing the MNP size, increasing the ETTI by increasing their areal density, and increasing the work function difference between the metal electrode and MNPs should result in a more enhanced barrier modulation effect. However, no concise analytical description of the ETTI model has been proposed so far except for a few reported numerical simulations^11^. None of the reported literature to our knowledge has shown complete symmetric Ohmic behaviour after embedding MNPs into metal-low doped semiconductors contacts^12^,^13^,^14^,^15^,^16^,^17^. Kishore *et al.* studied contact modification of Ti/n-Ge and Ti/p-Ge by Au NPs with diameter around 10.4 ± 0.7 nm. For Ti/n-Ge, ~1000 times of reverse saturation current density improvement from ~0.01 to ~10 A/cm^2^ were observed^16^. However, it is still ~9 times lower than the forward bias current density of ~90 A/cm^2^, indicating a quasi-Ohmic contact instead of ideal Ohmic contact. Though improvement in reverse saturation current density were observed for metal contact on n-type semiconductor, the improvement for contact on p-type semiconductor was marginal^16^,^17^. Gorji *et al.* reported the use of Au NPs to reduce the contact resistance of metal contact on both moderate to low doped p-Si and n-Si (resistivity: 0.01–30 Ω cm)^17^. For Al/n-Si, ~100 times of current density improvement from 0.001 to 0.1 A/cm^2^ were observed. However, the forward and reverse current density are asymmetric with ~100 times different. For the Al/p-Si sample, the marginal improvement was observed with the help of Au NPs. Note that the MNPs used in previous studies with the purpose of SBH modulation are also relatively larger sized (compared to MNPs utilized in this study), and the techniques used to deposit them had limited control over their size distribution, areal density, and coverage rate, which can limit its application in next generation device with nanometer-scale contacts^15^,^16^,^19^,^20^. A thorough study on the effect of barrier modification due to the incorporation of sub-nm sized MNPs, the combined effect of both thin dielectric and MNPs, and the role of Coulomb Blockade and Quantum confinement has not been reported. A solid experimental result with significant improvement in contact resistance, especially for low doped p-type semiconductor, has not been reported.

In this study, we reduce the M-S junction barrier by incorporating high work function sub-2 nm Pt NPs as current injection hotspots and a thin dielectric layer to partially depin the Fermi level. These ultra-fine NP with high density and narrow size distribution are fabricated by a Tilted-Target-Sputtered (TTS) deposition technique^21^,^22^,^23^,^24^,^25^,^26^. Two different types of metals (Ti and Au) were used as electrodes to study these effects on converting both Schottky contacts (Ti/p-Si) and poor Ohmic contact (Au/p-Si) into good Ohmic contact, respectively. We report the properties of the size-dependent Pt NPs and their role in achieving Ohmic contact at the M-S interface with either a 0.98 nm Al_2_O_3_ or a 1.6 nm SiO_2_ dielectric layer, or both. In contrast to most other works where relatively low work function MNPs were used, Pt was chosen to maximize the work function difference between the metal electrode and the NPs so as to maximize the localize electric field of the surface dipoles. The thin dielectric layers were added to partially depin the Fermi level at the interface especially the region not covered by the Pt NPs; these layers were also added to control and study how the metal-metal NP or metal NP- Si interface dipole affect the barrier reduction and achieve Ohmic contact. As the sub-2 nm MNPs are embedded into dielectric layers between the MS contact, the electron addition energy of the incorporated MNP matrix, which comprises of Coulomb charging and quantum confinement energy have been taken into consideration. A significant improvement over other previously reported work has been obtained^17^. We further show that the contact can be modulated to be either Schottky or Ohmic using the same gate metal, albeit varying only the embedded Pt NP size and its areal density.

## Device Fabrication

**Figure 1:**
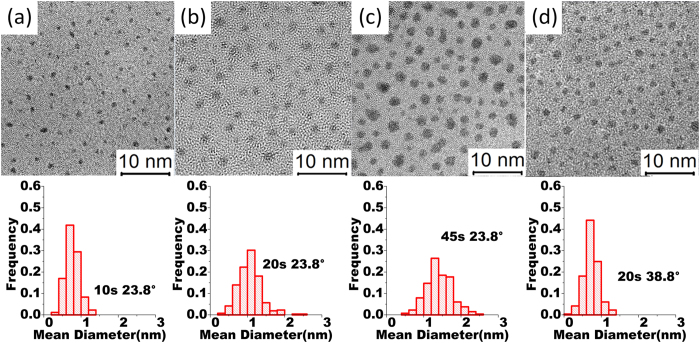
TEM image of TTS-deposited Pt NPs with deposition conditions of (**a**) 10s 23.8° LD, (**b**) 20s 23.8°, (**c**) 45s 23.8°, (**d**) 20s 38.8° HD and their corresponding size distribution. Adapted from ref. 21.

Metalorganic contaminants were removed from low-doped p-type Si (p-Si) using a modified Shiraki cleaning process described in^27^. For some of these cleaned samples the chemically grown SiO_2_ obtained after immersing them in HCl:H_2_O:H_2_O_2_ was preserved by without doing the last step of HF:CH_3_OH soak. The thickness of this SiO_2_ layer was measured to be 1.61 ± 0.24 nm. An AJA International ATC 2000 magnetron sputtering system was used to deposit Pt NPs. A two-inch Pt target with 99.99% purity from Kurt J Lesker was mounted on a sputtering gun which can be tilted at an angle with respect to the substrate in the horizontal plane. The chamber was evacuated to 10^−8^ Torr, and the Pt target was pre-sputtered for 10 min at an RF deposition power of 100 W before the actual deposition. The substrate holder was configured as the anode (grounded). Pt NPs were deposited with a high purity Ar gas (99.999%) with a flow rate of 10 sccm, at a working pressure of 4 mTorr, the ambient temperature of ~300 K, six-inch target to substrate distance and 20 rpm substrate rotation speed. A 30 W RF power (13.56 MHz) was used to sputter Pt NPs with the deposition time and target angle tuned to obtain different sizes and areal densities. Samples were placed at the centre of the substrate holder to minimize the influence of radial displacement of the grids on the observed nanoparticle characteristics. The Pt NPs were characterized using a high-resolution transmission electron microscope (HRTEM)-Tecnai F20 (200 kV) with beam shift capabilities. For image characterization, a 5 nm amorphous Al_2_O_3_ thin film was deposited on a holey carbon film grid using a Kurt J Lesker AXXIS electron beam evaporator at room temperature before deposition of the Pt NPs (See ref. 21 for more details). Same masks used later on for patterning the metal electrodes were also used for patterning the Pt NPs to prevent lateral tunnelling current between electrodes. Deposition times of 10, 20, and 45 s at 23.8° target angle corresponded to Pt NP sizes of 0.74 ± 0.12 nm, 1.11 ± 0.28 nm, and 1.45 ± 0.36 nm (based on HRTEM analysis), respectively. With larger target angle (38.8°) and deposition time of 20 s, we achieve Pt NP size of 0.72 ± 0.12 nm which is similar to the 0.74 ± 0.12 nm size of the 10 s 23.8° Pt NPs, except for the higher areal density of (1.09 ± 0.06) × 10^13^/cm^2^ (0.72 nm high density (HD)) compare to the (7.08 ± 0.30) × 10^12^/cm^2^ of the 10 s 23.8° Pt NPs (0.74 nm low density (LD)). (see ref. 21, 22, 23,25 for details). The TEM images of different size of TTS-deposited Pt NPs and the size distribution analysis were reported in^21^ and shown in Fig. 1. The observed characteristics for Pt NPs used in this study are summarized in Table 1. We have performed thermal stability test for capped (3 nm Al_2_O_3_) and uncapped ~1.55 nm size Pt NPs. Based on the TEM study, no significant diffusion or coalescence is observed for uncapped Pt NPs with annealing temperature up to 400 °C. For capped Pt NPs, they are stable up to 950 °C, indicating a successful prevention of the Pt NPs from diffusion and coalescence by the 3 nm Al_2_O_3_ capping layer. For the Pt NPs capped with the 0.98 nm Al_2_O_3_ used in this study though it has undergone stability test and it may not provide as good diffusion prevention as the 3 nm one, it is reasonable to expect a higher temperature stability than the uncapped one (See supplemental information for more details). A 0.98 ± 0.13 nm Al_2_O_3_ layer was then deposited by Atomic Layer Deposition (ALD). ALD was performed on an S200 system (Cambridge NanoTech) using deionized water (DI) and trimethylaluminum (TMA) acquired from Sigma-Aldrich (St. Louis, MO). After loading the samples, a continuous flow of 5 sccm N_2_ was passed through the chamber. The heaters for the chamber and lid O-ring were set to both 150 °. After four hours, the N_2_ flow rate was increased to 20 sccm. Water was then cycled 20 times to saturate the sample surface with –OH bonds before deposition using a 20 ms water pulse followed by 8 seconds N_2_ purge flow. The deposition was carried out using 12 cycles of the following flows: 20 ms DI water pulse, 8 second N_2_ purge, 15 ms TMA vapor pulse, and 8 second N_2_ purge. After deposition, the N_2_ flow rate was reduced to 5 sccm, and the chamber was cooled to 65 °C before removing the samples. After the oxide and Pt NP growth, 40 nm of Ti or 80 nm Au metal contact was then deposited on top of the samples using e-beam evaporation. For the Ti contact, an additional 40 nm of Au layer were deposited covering the Ti layer to prevent its oxidation. The backside of the substrate was then coated with Cr/Au to form a good Ohmic back contact using e-beam evaporation. A 2 nm of Cr layer was deposited as an adhesion layer before the 80 nm Au. Samples were then loaded in an e-beam chamber under high vacuum conditions (~10^−8^ Torr) and annealed by flowing 30 SCCM of H_2_ at 260 °C for 1 hour. With the shutter of the cryopump switched on, the chamber pressure raised to ~10^−3^ Torr with the introduction of the H_2._ The H_2_ annealing was used to improve the silicon/metal interface and oxide quality. All the oxide thickness were measured by using a variable angle spectroscopic ellipsometer (VASE, J.A. Woollam, Inc., USA). The devices were characterized in a Janis ST-500 probe station system which has the capability of achieving vacuum (down to ~5 mTorr) and can be cooled down to 80 K. Current-Voltage (I–V) and Capacitance-Voltage (C–V) characteristics were measured by a Keithley 4200 SCS system. About 7–10 devices for each condition were measured and plotted with error bars, where applicable.

Different device structures and configuration were fabricated and studied within the scope of this study (more details of which are provided in the results and discussion section). With the device structure of [Ti/Oxide/p-Si], we first discuss the role of the thin dielectric layers (SiO_2_ and Al_2_O_3_) in barrier height reduction. Later, using the structures of [Ti/Al_2_O_3_/Pt NPs/p-Si], [Ti/Pt NPs/SiO_2_/p-Si], and [Au/Pt NPs/SiO_2_/p-Si], we discuss the role of introducing Pt NPs, the effect of different Pt NPs/dielectric stacking configurations and the influence of different gate metals. Lastly, we discuss the effect of the size and density of Pt NP embedded between SiO_2_ and Al_2_O_3_ thin oxide in barrier modification using the configuration-[Ti/Al_2_O_3_/Pt NPs/SiO_2_/p-Si]. All the device conditions studied in this work are also summarized in Table 2.

## Results and Discussions

### Role of thin dielectric layers (SiO_2_ and Al_2_O_3_) in barrier height reduction

The reason for choosing 0.98 nm Al_2_O_3_ is based on the theoretical study of Roy *et al.*’s work on the effect of fix charges and thin dielectric thickness to the Fermi-level depinning^7^. According to their observation for 0 to 3 nm thin dielectric layers, 1 nm thick Al_2_O_3_ or Si_3_N_4_ is expected to have better fermi-level depinning than HfO_2_ and TiO_2_. In our previous works, the optimized thin ALD Al_2_O_3_ as dielectric layers of Si-based MOS capacitors showed small hysteresis window (~30 mV), indicating low mobile charge and low Al_2_O_3_-Si interface trap sites^23^,^25^,^28^, which is important for device application. The effect of the thin SiO_2_ is also of interest for a metal-Si system as a thin SiO_2_ may present on a Si surface after exposure to the atmosphere for some time.

**Figure 2:**
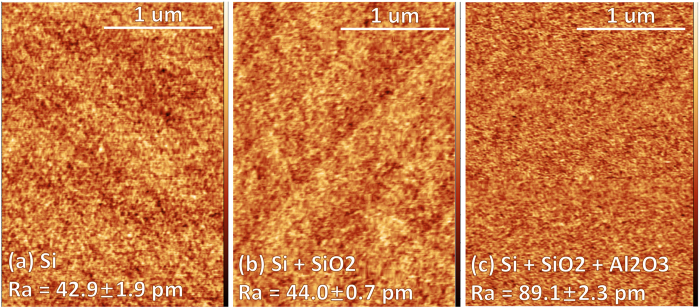
AFM measurements showing the morphology of different surfaces: (**a**) Si, height scale: 0.10–0.61 nm; (**b**) SiO_2_, height scale: 0.03–0.60 nm ; and (**c**) Al_2_O_3_, height scale: 0.03–1.16 nm.

Figure 2 compares the AFM surface morphology of the Si, SiO_2_ and the Al_2_O_3_ surface, respectively. The surface roughness of the chemically growth SiO_2_ surface is 44.0 ± 0.7 pm which is within the error bar of 42.9 ± 1.9 pm of the Si after removing the SiO_2_. The roughness was increased to 89.1 ± 2.3 pm after growing the 0.98 nm Al_2_O_3_ on the SiO_2_. Noteworthy that even the AFM data show quite small surface roughness on all 3 surfaces. For ALD Al_2_O_3_ thinner than 2 nm, there may be pinholes which we were able to verify by using cyclic voltammetry. For the contact application, these 0.98 nm Al_2_O_3_ may not have to be completely pinhole free, because the overall contact behavior can be similar to the dominant surface condition. Since the Al_2_O_3_ is used to partially depin the Fermi level of the metal from the neutral level, the present of any pinholes can just reduce this depinning effect. If the density of pinholes is far less than that of the Pt NPs, it may not have a significant impact on the below discussion.

**Figure 3:**
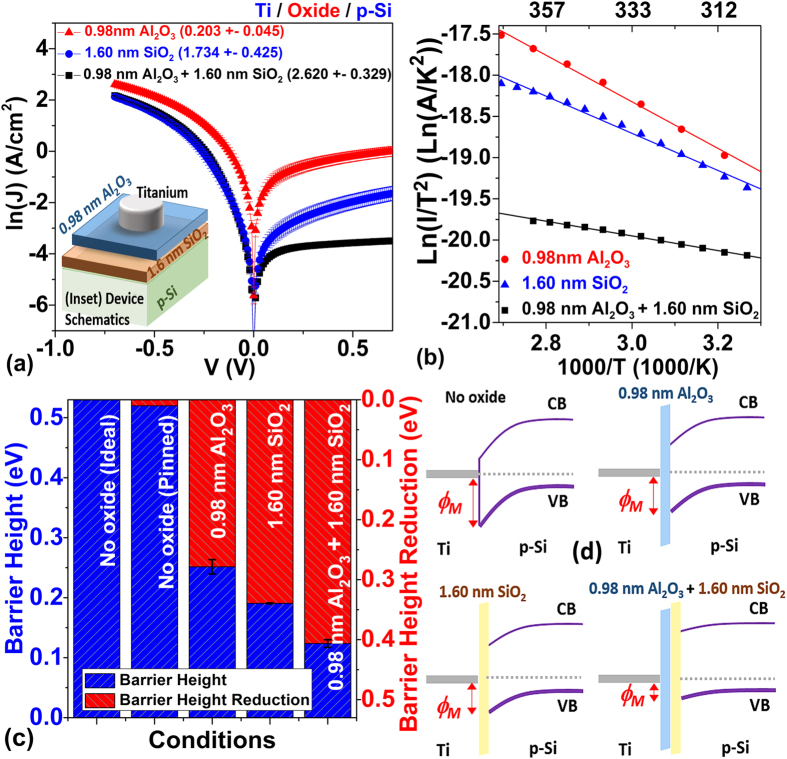
Comparison of Ti-p Si contact with different thickness/type of dielectrics (**a**) Ln(J)-V characteristics. The brackets give the specific contact resistivity. Inset: Schematics of device structures; (**b**) the Richardson plots; (**c**) the barrier heights; (**d**) schematics of energy band diagram showing the barrier reduction of Ti-p Si contact by insertion of thin dielectrics.

When inserting a dielectric layer, it must be noted that although the SBH is lowered due to Fermi level depinning of the oxide, the effective barrier width for tunnelling is increased when taking into consideration the contribution of the insulating oxide barrier itself. Thus, for the purpose of reducing specific contact resistivity, the oxide thickness must be optimized to obtain a favourable trade-off between the barrier height and effective barrier width. Previous studies have reported that the optimized thickness for Al_2_O_3_ to reduce the specific contact resistivity effectively, is ~1 nm^7^,^8^. The Ti-p-Si system forms a Schottky contact with an experimental SBH of ~0.5 eV. A thin dielectric was inserted between Ti and Si to specifically ‘depin’ the Fermi level formed due to metal-induced gap states or interface defect states. Devices with 0.98 nm atomic layer deposited (ALD) Al_2_O_3_ were used as a control and compared with devices comprising of 0.98 nm Al_2_O_3_, or 1.6 nm chemically growth SiO_2_, or both. Devices with 0.98 nm Al_2_O_3_ showed the highest current density at both positive and negative bias (Fig. 3(a)). The lower current injection of other conditions was due to the increase of tunnelling resistance with thicker oxide, which offsets the benefit of SBH reduction.

Utilizing the thermal emission (TE) model^1^, one can analyse the Schottky barrier properties through *J-V* characteristics in the forward bias direction. For moderately doped semiconductors, the *J-V* characteristics in the forward direction is given by Eq. (1)).


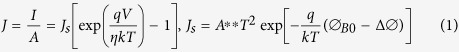



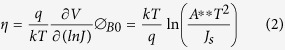


where *J*_*s*_ is the saturation current density, A is the device area, A^**^ is the effective Richardson constant, (which has been reported to be ~30 for p-Si^1^), T is the temperature in Kelvin, q is the electron charge 1.6 × 10^−19^ C, k is Boltzmann’s constant 1.38 × 10^−23^ J/K, ∆Φ is the image force lowering, and V is the forward bias voltage. *η* and *Φ*_*BO*_ are the ideality factor and barrier height which can be calculated by Eq. (2)). The ∂*V*/∂(*lnJ*) term in the ideality factor equation is the reciprocal of the slope in the linear fitting of the *J-V* characteristic and *J*_*s*,_ is the intersection of the linear fit of the J-V characteristic at the y-axis. In other words, upon obtaining the intercept and slope by linear fitting the *J-V* characteristic, *Φ*_*BO*_ and *η* can be calculated. When *η* = 1, the carrier transmission is dominated by TE, while when *η* > 1, the main transmission mechanism involves field-emission (FE) or thermal-field-emission (TFE).

By using Eq. (1) and (2), the ideality factor-of ~1.3–1.6 was obtained, which meant that FE or TFE should be taken into consideration for the current transport mechanism. The specific contact resistivity for all samples are calculated using *ρ* = ∂*V*/∂*J* (*V* = 0) and summarized in Table 3.

For further analysis of the current transport behaviour, the activation energy measurements (J-V-T) were performed. According to^1^,


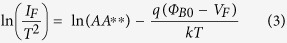


where *q*(Φ_*B*0_ − *V*_*F*_) is considered to be the activation energy. Over a limited range of temperatures (typically around room temperature), the value of A** and Φ_*B*0_ are essentially temperature independent. Thus for a fixed forward bias *V*_*F*_, the slope of a plot of In(I_F_/T^2^) versus 1/T yields the barrier height Φ_*B*0_, and the ordinate intercept at 1/*T* = 0 yields the product of the electrically active area *A* and the effective Richardson constant A**. The slopes of the curves in the Richardson plots (Fig. 3(b)) were used to calculate the barrier heights (Fig. 3(c)). It’s seen that devices combining both the Al_2_O_3_ and the SiO_2_ layers have lowest barrier heights (~0.12 eV), which indicates better Fermi level depinning.

The energy band diagrams for different structures demonstrating this behaviour were plotted in Fig. 3(d). In the next two sections, we report the modulation of the contact by embedding different size/density of TTS deposited Pt NPs in these devices.

### Role of introducing non-isolated Pt NPs and the effect of different stacking configuration of Pt NPs layer and the thin dielectric layer on modifying Shottky and Ohmic contact

**Figure 4:**
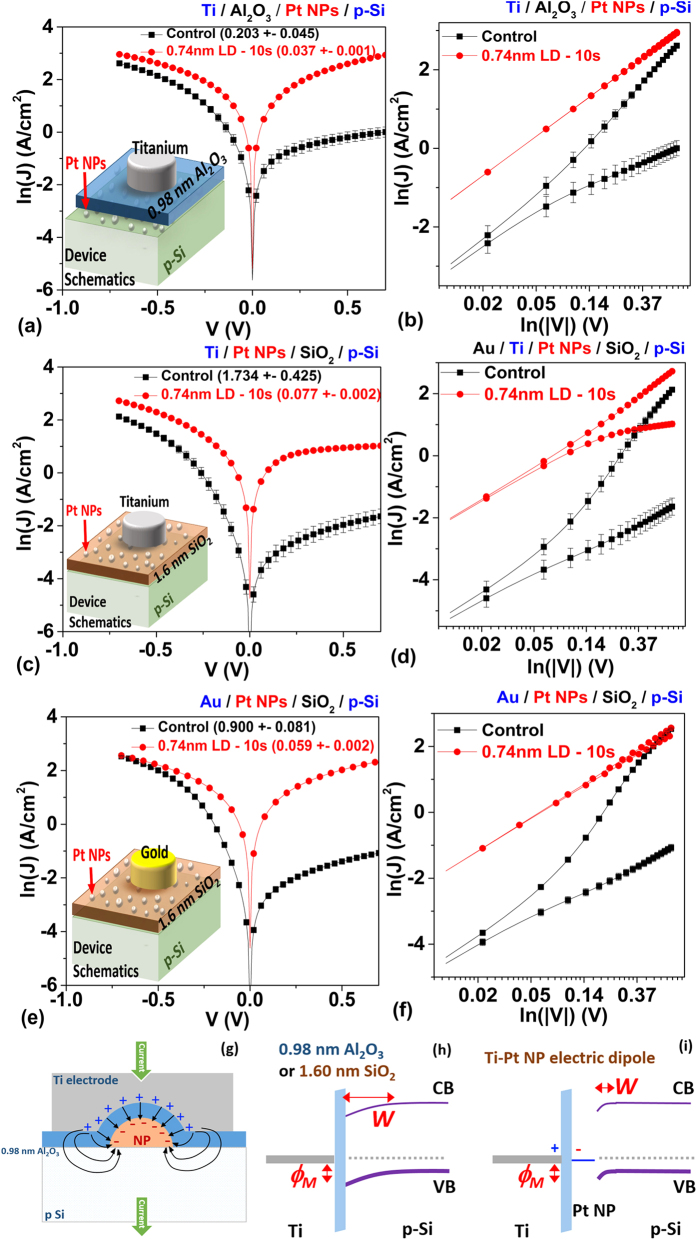
Comparison of Ln(J)-V characteristics for (**a**) Ti-Al_2_O_3_-p Si contact with and without 0.74 nm LD Pt NPs between the Al_2_O_3_ and Si surface; (**c**) Ti-SiO_2_-p Si contact with and without 0.74 nm LD Pt NPs between the Ti electrode and SiO_2_ surface; (**e**) Au-SiO_2_-p Si contact with and without 0.74 nm LD Pt NPs between the Au electrode and SiO_2_ surface; Inset: Schematics of the device structures. (**b**,**d,f**) The corresponding Ln(J)- Ln(|V|) characteristics of (**a**,**c**,**e**,**g**) schematics showing the dipole formation between Ti electrode and Pt NP; (**h,i**) schematics of energy band diagram showing the reduction of depletion width and barrier height with the present of the Pt NPs.

Figure 4(a–f) show the J-V characteristics of the device with and without Pt NPs in combination with different thin dielectric stacking and different macroscopic metal electrodes. Here, the use of the term ‘non-isolated’ essentially means that the introduced Pt NPs are in direct contact with either the top gate electrode or the p-Si substrate. The ‘non-isolated’ case is somewhat unique since the Pt NPs are in direct contact with either the top electrode or the bottom p-Si, there is no Coulomb blockade effect governing charge injection in these cases. In this study, two different types of metals (Ti and Au) were used as electrodes to explore the role of the non-isolated Pt NPs on modifying Schottky contacts (Ti/p-Si, Fig. 4(a–d)) and Ohmic contact (Au/p-Si, Fig. 4(e,f)), respectively. For the Ti/p-Si case, Ti was covered with a 40 nm layer of Au to prevent Ti from oxidation.

Figure 4(a) compares the ln(J)-V characteristics of the Ti/Al_2_O_3_/p-Si contact with and without embedded 0.74 nm LD Pt NPs. The layer of Al_2_O_3_ or SiO_2_ is found to be beneficial to Fermi level depinning and SBH reduction according to the first section of Results and Discussions. The addition of Pt NPs was found to enhance further the current injection for both forward and reverse bias. In fact, there was magnitude 18 folds improvement in the reverse current density from ~1 A/cm^2^ to ~18.7 A/cm^2^. The ln(J)-ln(|V|) curve in Fig. 4(b) further confirmed the good linearity and completely symmetric current conduction for both forward and reverse region (current ratio of 1.041 @ ± 0.7 V) – an ideal Ohmic contact was thus formed with this configuration.

Comparing the J-V characteristics displayed in Fig. 4(a–d) for devices with Ti gate electrode, we also found that samples with Pt NP with no dielectric layer directly on the p-Si surface have overall higher current density, compared to the device with a dielectric layer directly on top of p-Si surface, especially in reverse bias region and lower contact resistivity. This observation can be explained by the partial potential drop within the thin dielectric and the lowering of dipole induced electric field to the semiconductor, weakening the SBH modulation due to the Pt NPs.

By comparing J-V characteristics displayed in Fig. 4(c–f) it can be surmised that the introduction of Pt NPs on top of the thin SiO_2_ layer converted both Ti-SiO_2_-p Si contact and Au-SiO_2_-p Si contact from SB contact into quasi-Ohmic contact, with ~10^4^ improvement in reverse current density for both cases. In particular, Au-Pt NP-SiO_2_-p Si contact shows linear and symmetric Ohmic behavior. Overall, about 10 ~ 20 times reduction of specific contact resistivity were observed for both Schottky and Ohmic contact devices (Table 3). Note that the current level of the Au-SiO_2_- p Si sample is quite low compared to a good Ohmic contact. This can be explained by the poor adhesion of Au on Al_2_O_3_, resulting in either an increase of equivalent tunneling barrier or reduction of the effective electrically active area of the electrode, as discussed in ref. 1.

Metal-Pt NP dipole enhanced electric field was determined to be the main cause of the barrier modification and current modulation (Fig. 4(g)). When a combination of different metal patches are brought into contact with a semiconductor in the case of a large area electrode (where electrode contact area and semiconductor depletion width are much larger than the diameter of the Pt NPs) the macroscopic current transport cannot be modelled by a parallel combination of these different SB MS contacts. Instead, the electric field induced by the work function difference of the metal and Pt NPs tends to modulate the local SB and thereby to modulate the current transport. The schematics of the energy band diagram in Fig. 4 (h–i) illustrates this effective reduction of SBH and depletion width of a macroscopic MS contact with embedded MNPs.

**Figure 5:**
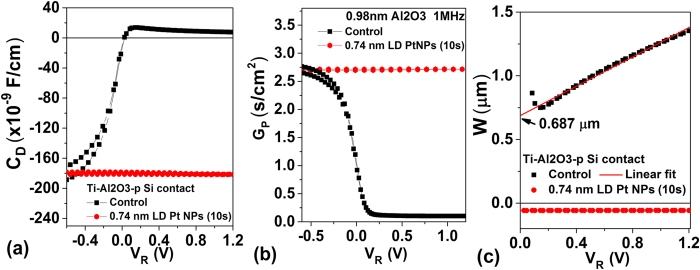
Comparison of (**a**) C_D_-V, (**b**) G_P_-V, and (**c**) depletion width Ti-Al_2_O_3_-p Si contact with and without 0.74 nm LD Pt NPs (10s).

Capacitance-Voltage (C–V) measurements were also performed to study the barrier modification. The depletion width of the semiconductor under reverse bias can be estimated by,


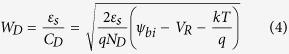


where *ε*_*s*_ is the permittivity of the semiconductor, *N*_*D*_ is the doping concentration, *ψ*_*bi*_ the conduction band minimum (CBM) band bending at zero bias, and *V*_*R*_ the reverse bias voltage (in the positive voltage range for our cases). For Ti/0.98 nm Al_2_O_3_/p-Si sample, the depletion width at zero bias can be calculated to be ~685 nm using (4). For Ti/0.98 nm Al_2_O_3_/0.74 nm LD Pt NPs/p-Si sample showing Ohmic behavior, C_D_ is a negative number (Fig. 5(a)), which is an indication of high parallel conductivity (Fig. 5(b)) due to the current injection from the Pt NP hotspots, which tends to dominate the overall reactance (represented as negative capacitance), and hence the depletion region formed at the p Si band edge cannot be determined by conventional C–V method (Fig. 5(c)). Further circuit analysis correcting the measured C_D_ is required to make C–V analysis useful for the studied Pt NPs embedded samples displaying high conductivity.

For an “ideal” MS Schottky barrier (e.x. excluding the effect of image force lowering, and interface impurity), if V_P_(z) represents the CBM potential reference to the Fermi level, with z being the depth into the MS interface and W being the width of the depletion region, then for 0 < z < W,


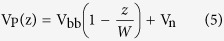


where V_bb_ = *ϕ*_*B*0_ − *V*_*n*_ − *V*_*a*_ − *V*_*o*_ is the total band bending at a given applied bias *V*_*a*_ for a SBH of *ϕ*_*B*0_, *V*_*o*_ the SBH lowering due to a thin dielectric layer between the MS interface, and *V*_*n*_ is the difference of the CBM and the Fermi level of bulk semiconductor. The maximum value of V_P_(z) yields the SBH (max [V_P_(z)]). According to Tung’s^10^ ISBH model, by introducing the potential due to a planar circular metal-metal interface dipole layer with a moment per area of 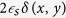 and radius of R into a MS contact with mean SBH of *ϕ*_*B*0_, Eq. (5) can be modified as follows to represent the V_P_(z) at the centre of the circular patch along the z axis:


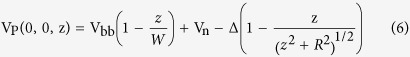


with Δ = *ϕ*_*B*0_−*ϕ*_*B*,*patch*_ being the SBH difference between the two metals before contact. By differentiating Eq. (6) with respect to z, the electric field is given by


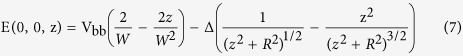


**Figure 6:**
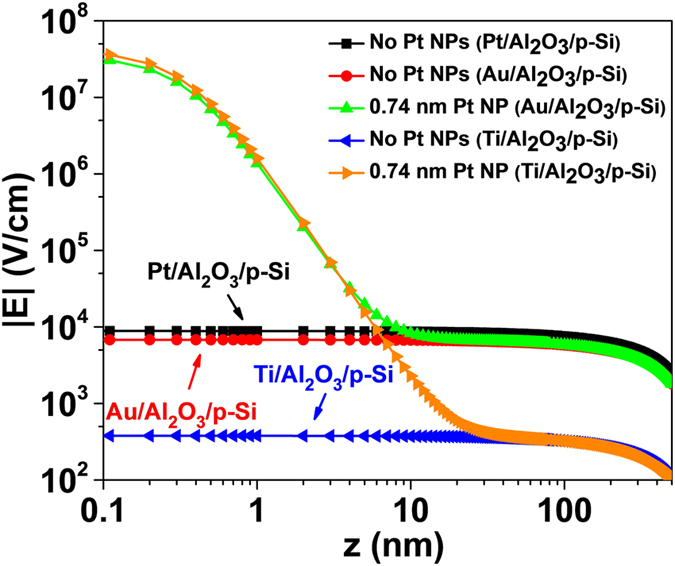
Comparison of simulated E-field enhancement in z direction for Ti/Al_2_O_3_/p-Si and Au/Al_2_O_3_/p-Si contacts with and without 0.74 nm size Pt NPs.

A simulated electric field (E) vs. depth of the contact (z) for Ti/Pt NP/p-Si and Au/Pt NP/p-Si system using Eq. (7) were plotted in Fig. 6. Large E-field enhancement was seen for the sample with embedded small sized Pt NPs, which is qualitatively in good agreement with the observed J-V characteristics of Fig. 4(a) as Pt with much higher work function than Ti yields a large ideal SBH difference of Δ. The larger SBH difference between Pt and Ti compared to that between Pt and Au resulted in larger E-field enhancement as can be seen in the plotted E-field enhancement w.r.t. *z* reported in Fig. 6. The Pt NPs with *R* as small as 0.37 nm are expected to induce a large electric field near the MS interface, resulting in reduction of local SBH and thereby facilitating current injection at these hot spots. For sample with 1.6 nm SiO_2_ below the Pt NPs, one may replace “z” to “z-1.6 nm” in the second term of Eq. (7), which qualitatively yields the same aforementioned conclusion of the E-field enhancement by Pt NPs.

### Role of size and density of isolated Pt NP embedded between SiO_2_ and Al_2_O_3_ thin oxide in contact modification of both Schottky and Ohmic contact

The devices consisting of ‘isolated’ embedded NPs between two dielectric layers [0.98 nm Al_2_O_3_/Pt NPs/1.6 nm SiO_2_/p-Si] are reported in this section. By controlling the deposition time and target angle, different size and density of Pt NPs were deposited (Table 1). In contrast to devices studied in the previous section, with both the top electrode and the bottom p-Si being electrically isolated from the Pt NPs by a thin dielectric, Coulomb blockade effect must be taken into consideration in addition to the field enhancement in these cases. Two different types of metals (Ti and Au) were used as electrodes to study the role of the dielectric embedded Pt NPs on modifying Schottky contacts (Ti/p-Si) and Ohmic contact (Au/p-Si).

**Figure 7:**
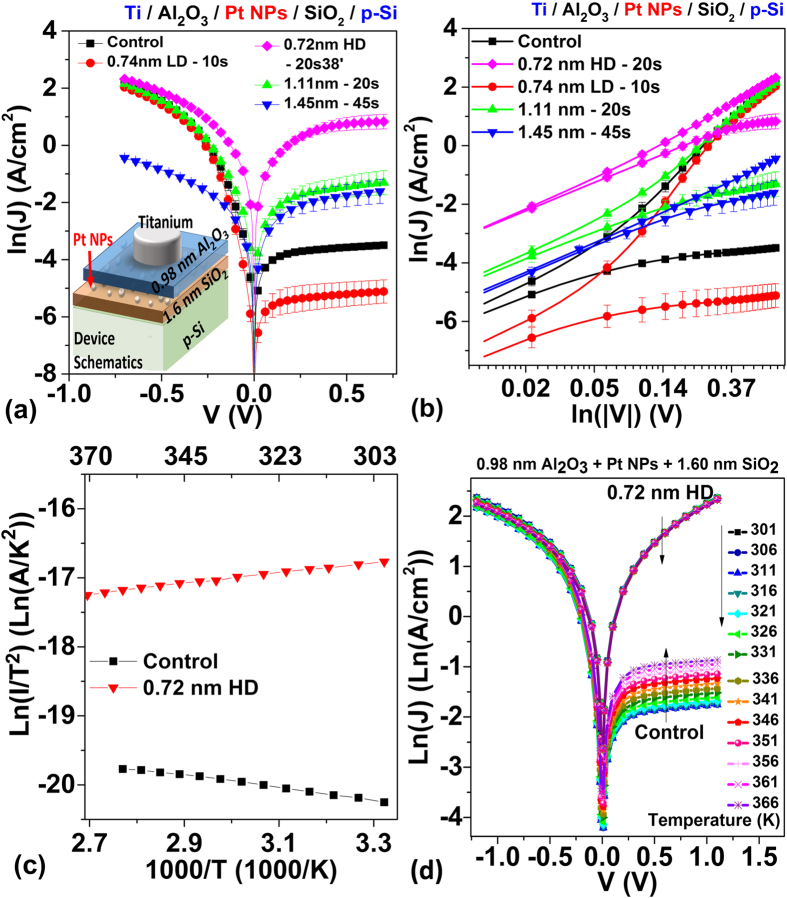
Comparison of Ti-p Si contact with different size of Pt NPs sandwich between the 0.98 nm Al_2_O_3_ and 1.6 nm SiO_2_ (**a**) Ln(J)-V characteristics. Inset: Schematics of device structures; (**b**) Ln(J)- Ln(|V|) characteristics; (**c**) the Richardson plots. (**d**) Ln(J)-V-T characteristics.

For these studied theoretical Schottky contact (Ti/p-Si) devices, significant Pt NP size-dependent J-V characteristics were observed in Fig. 7(a,b). With embedded 0.74 nm LD Pt NPs, more imbalance in current injection—particularly ~2 order of magnitude lowering of reverse bias current was observed compared to the control sample. While tuning the diameter of the Pt NPs from 0.74 nm to 1.11 nm, the reverse bias current improved by more than two order of magnitude compared to the control. Further increasing the size of the Pt NPs to 1.45 nm resulted in a decrease in forward bias current compared to the sample with embedded 1.11 nm Pt NPs. The 0.72 nm HD sample yields the best current improvement in both forward and reverse polarity, and the lowest specific contact resistivity of 0.165 ± 0.019 Ω-cm^2^ was achieved in this configuration (compared to 2.620 ± 0.329 Ω-cm^2^ of the control sample (Table 3)). Although the Pt surface coverage rate for samples with the 0.72 nm HD Pt NPs is only 4.570 ± 0.057%, ~3 times lower than that of samples with 1.45 nm Pt NPs (12.764 ± 1.309%), the specific contact resistivity is, however, ~10 times higher. To conclude, ~26 times improvement in specific contact resistivity with 1.45 nm Pt NPs compared to the control sample without embedded Pt NPs was observed.

When trying to determine the barrier height reduction due to 0.72 nm HD Pt NPs, a small negative slope was observed in the measured Richardson plots (Fig. 7(c)), in contradiction to the positive slope of the control sample. This can be explained by the reduction of the barrier height and the contact resistance by the Pt NPs, resulting in weak inverse dependence of the current density to the temperature ((Fig. 7(d)).

The measured reverse saturation resistance of the device *R*(*T*) = *R*_*Ti*_ + *R*_*top contact*_ + *R*_*Si*_ + *R*_*bottom contact*_ + *R*_*Au*_ The resistivity for titanium (*R*_*Ti*_) or gold (*R*_*Au*_) is usually in the order of 10^−5^ Ω cm at 100–400 K which is much smaller than that of Si (*R*_*Si*_ ~10^−1^−10^1^ Ω cm) with doping concentration of 1.5 × 10^15^ cm^−3^. The bottom contact resistance (*R*_*bottom contact*_) is usually negligible due to the large area of the bottom of the whole substrate being the contact area. So *R*(*T*) ≈ *R*_*top contact*_(*T*) + *R*_*Si*_(*T*). For the control sample with SB contact, the large *R*_*top contact*_ (*T*) dominates the overall *R*(*T*), resulting in current density positively correlated to the temperature which is a typically characteristic for SB contacts. For Pt NP modified samples with reduced SBH, the contact resistance become comparable or smaller than the resistance of bulk silicon. The temperature dependent characteristics of *R*(*T*) become combination of *R*_*top contact*_ (*T*) and *R*_*Si*_(*T*). The silicon resistivity is given by *ρ*_*Si*_ = (1)/(*qμ*_*p*_*p*), where *μ*_*p*_ is the hole mobility and *p* is the hole density^1^,^29^,^30^. 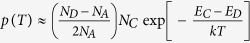 is weak correlative to *T* and remains almost constant in the range of 150–400 K, while for low doped Si, *μ*_*p*_(*T*) ∝ (1)/(*T*^3/2^) decreases as *T* increases; overall, *ρ*_*Si*_(*T*) increases as *T* increases. For the case of the measured 0.72 nm HD sample, *R*(*T*) become weak positive correlated to *T*. Similar temperature dependent behaviour is expected for the “nonisolated” Pt NPs samples.

**Figure 8:**
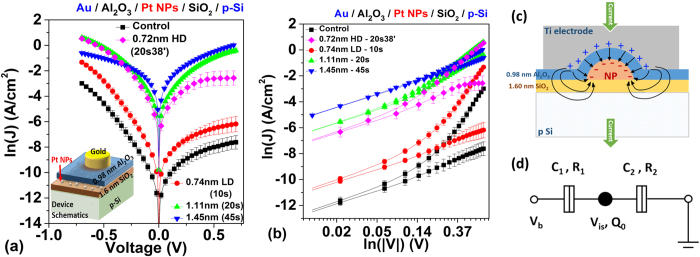
Comparison of Au/p-Si contact with different size of Pt NPs sandwich between the 0.98 nm Al_2_O_3_ and 1.6 nm SiO_2_ (**a**) Ln(J)-V characteristics. Inset: Schematics of device structures; (**b**) Ln(J)- Ln(|V|) characteristics; (**c**) schematics showing the dipole formation between Ti or Au electrode and Pt NP; (**d**) Equivalent circuit of the device showing the Coulomb blockade effect.

For the studied Ohmic contacts (Au/p-Si) devices, Pt NP size-dependent J-V characteristics are presented in Fig. 8(a,b). The current level of the control sample, however, is significantly lower than expected, indicating a poor Ohmic contact. This can be explained by the poor adhesion of the Au on the Al_2_O_3_, resulting in either an increase of equivalent tunneling barrier or reduction of effective electrically active electrode area^1^. However, with the assistance of the embedded 1.45 nm Pt NPs, the specific contact resistivity can be significantly reduced ~1600 times from 2485.409 ± 532.769 Ω-cm^2^ to 1.543 ± 0.149 Ω-cm^2^.

In this ‘isolated’ embedded Pt NP case, there are mainly two NP-related mechanisms responsible for the current modulation observed in Fig. 7(a,b): (1) Enhanced electric field due to Ti-Al_2_O_3_-Pt NP interface dipole, resulting in the SBH modulation (Fig. 8(f,g)) and the potential pinch-off effect; (2) Current suppression due to NP size-dependent Coulomb charging energy (CCE) and quantum confinement energy (QCE)^31^. The first effect is identical to the “nonisolated” case and has been explained in the previous sections. For the second effect, a high CCE or QCE typically means the carriers are blocked under low voltage condition, and a large enough voltage is required before the carriers can tunnel through the NPs. Under this condition, although the carriers can still tunnel through the region without the NP, the overall current level of the device may still be suppressed.

The CCE of an NP can be estimated by the equivalent circuit given in Fig. 8(g),


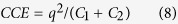


where *C*_1_ and *C*_2_ are the junction capacitance of the Ti/0.98 nm Al_2_O_3_/Pt NPs and Pt NPs/1.6 nm SiO_2_/p-Si respectively. The QCE can be estimated by *QCE* = 2 * *E*_F_/(3 * *N*), where *E*_*F*_ is the Fermi energy of Pt and *N* is the total number of electrons for a Pt NPs. The total electron addition energy (EAE) can then be calculated by *EAE* = *CCE* + *QCE.* Note that due to the asymmetric capacitance and resistance of the two tunnelling junctions, the voltage required for charging the Pt NPs from the top (V_1_) or bottom (V_2_) electrodes will also depend upon the polarity of the gate bias, with


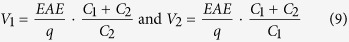


For the 0.74 nm LD sample with low areal density (7 × 10^12^ /cm^2^), the high EAE (547.5 meV compared to 147.2 meV for 1.45 nm Pt NP sample) was dominant over other mechanisms. Hence, the measured current was the lowest. Increasing the areal density to 1.1 × 10^13^ /cm^2^ (0.72 nm HD) resulted in a reduction of charging energy due to smaller inter-NP distance and stronger inter-NP coupling, considering the whole Pt NP layer as the tunneling matrix^31^. For this case, the effect of electric field enhancement of Ti-Al_2_O_3_-Pt NP interface dipole becomes more dominant and yields the highest current density in both reverse and forward bias. The interparticle coupling energy (ICE) is defined as a tunnelling matrix element (*t*) between equivalent single-particle states in nearest-neighboring NPs


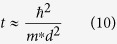


where *d* is the average interparticle distance and m^*^ is the effective electron mass of Pt. The ICE for 0.72 nm HD vs. 0.74 nm LD is 14.3 meV vs. 8.4 meV. The calculated CCE and ICE for each condition were summarized in Table 2.

For similar NP density, larger sized Pt NP embedded samples have a much lower charging energy, which facilitated the charge tunnelling, resulting in higher current injection level. SBH modulation due to the Ti-Al_2_O_3_-Pt NP interface dipole induced electric field was deemed as the dominant mechanism in this case. Note that the size of the Pt NPs play an important role in the SBH modulation similar to the case described in Eq. (7). It should be kept under consideration that the estimation of interface dipole induced localized electric field by ISBH model is, however, an underestimation compare to experimental results for ultra-small MNPs cases because it ignores the curvature of the interface dipole layer and treats it as a planar structure. For a more accurate determination of the dipole induced an electric field, the enhanced tunnelling at the metal-metal interface at the edge of the patches also needs to be taken into consideration^11^.

## Conclusion

We report M-S contact barrier modification through the introduction of sub-2 nm Pt NPs deposited by TTS, combining with a thin dielectric layer at the contact interface. Table 3 compares the reverse saturation current, forward/ reverse current ratio and specific contact resistivity for each condition studied in this work. For devices with non-isolated Pt NPs with a thin layer of dielectric (1.6 nm SiO_2_ or 0.98 nm Al_2_O_3_) on one side, the addition of Pt NPs improves the current injection significantly, regardless the type of metal (Au or Ti) used and the stacking order of the thin dielectric layer. For tested devices with Pt NPs embedded within the dielectric (isolated NPs), we found that the areal density and the size of the Pt NP played a significant role in subsequent current modulation. For similar NP density, larger sized Pt NP embedded samples have a much lower charging energy, which facilitated the charge tunnelling, resulting in higher current injection level. For devices with largest areal density (20s HD), the strong interparticle coupling significantly weakens the electron addition energy, and the effect of electric field enhancement of Ti-Al_2_O_3_-Pt NP interface dipole becomes more dominant and yields the highest current density in both reverse and forward bias. Table 4 summarized the device structures, NP conditions, reverse saturation current, and forward/ reverse current ratio of the metal/low doped p-Si contact of this work to the other published work. The maximum reverse saturation current density achieved by our low doped Si sample is 18 A/cm^2^, much larger than previous works with higher doping concentration^17^, and it is comparable to that of Ge-based devices which has much higher mobility than Si^16^. We attribute this improvement to be the much higher density of ultra-fine Pt NPs, its higher work function, and the combination effect of Pt NPs and a thin dielectric layer. Future work includes modelling these devices and incorporating the optimized sub-2nm Pt NP embedded structures at contacts for obtaining enhanced device and sensor performance.

## Additional Information

**How to cite this article**: Zheng, H. *et al.* Barrier Modification of Metal-contact on Silicon by Sub-2 nm Platinum Nanoparticles and Thin Dielectrics. *Sci. Rep.* **6**, 25234; doi: 10.1038/srep25234 (2016).

## Supplementary Material

Supplementary Information

## Figures and Tables

**Table 1:** Summary of the Pt NP conditions used in this study.

Conditions	Deposition time	Target Angle	NP diameter	Areal Density	Interparticle Distance
	sec	deg (°)	nm	10^12^ /cm^2^	nm
0.72 nm HD	20	38	0.72 ± 0.12	10.92 ± 0.62	2.31
0.74 nm LD	10	23	0.74 ± 0.17	7.08 ± 0.30	3.02
1.11 nm	20	23	1.11 ± 0.28	5.34 ± 0.48	3.22
1.45 nm	45	23	1.45 ± 0.36	7.73 ± 0.41	2.15

**Table 2:** Summary of the NP coverage rate, CCE, QCE, EAE, and ICE for each condition.

Pt NP diameter	Areal Density	Coverage Rate	CCE	QCE	EAE	ICE
Nm	10^12^ /cm^2^	%	(meV)	(meV)	(meV)	(meV)
0.72 ± 0.12	10.92 ± 0.62	4.57 ± 0.06	262.1	318.9	581.0	14.3
0.74 ± 0.12	7.08 ± 0.30	3.06 ± 0.02	253.8	293.7	547.5	8.4
1.11 ± 0.28	5.34 ± 0.48	5.17 ± 0.07	154.4	87.0	241.4	7.4
1.45 ± 0.36	7.73 ± 0.41	12.76 ± 1.31	108.2	39.0	147.2	16.5

**Table 3:** Comparison of the reverse saturation current, forward/ reverse current ratio and specific contact resistivity for each condition.

Top Electrode	Inter-contact Structures	Semiconductor	Type of NP	NP Diameter	Areal Density	Coverage Rate	Reverse Saturation Current Density	Forward/ Reverse Current Ratio	Specific Contact Resistivity
				nm	10^12^ /cm^2^	%	(A/cm^2^)		Ω cm^2^
Ti	Al_2_O_3_	p-Si	–	–	–	–	1.0022 ± 0.1940	13.62	0.203 ± 0.045
Ti	Al_2_O_3_/NPs	p-Si	Pt	0.74 ± 0.12	7.08 ± 0.30	3.05 ± 0.02	18.7460 ± 0.5866	1.03	0.037 ± 0.001
Ti	SiO_2_	p-Si	–	–	–	–	0.1934 ± 0.0535	43.37	1.734 ± 0.425
Ti	NPs/SiO_2_	p-Si	Pt	0.74 ± 0.12	7.08 ± 0.30	3.05 ± 0.02	2.7798 ± 0.2421	5.47	0.077 ± 0.002
Ti	Al_2_O_3_/SiO_2_	p-Si	–	–	–	–	0.0300 ± 0.0015	288.23	2.620 ± 0.329
Ti	Al_2_O_3_/NPs/SiO_2_	p-Si	Pt	0.72 ± 0.12	10.92 ± 0.62	4.57 ± 0.06	2.2946 ± 0.5831	4.44	0.165 ± 0.019
Ti	Al_2_O_3_/NPs/SiO_2_	p-Si	Pt	0.74 ± 0.12	7.08 ± 0.30	3.06 ± 0.02	0.0060 ± 0.0024	1262.69	10.663 ± 2.900
Ti	Al_2_O_3_/NPs/SiO_2_	p-Si	Pt	1.11 ± 0.28	5.34 ± 0.48	5.17 ± 0.07	0.2709 ± 0.1144	31.82	0.802 ± 0.140
Ti	Al_2_O_3_/NPs/SiO_2_	p-Si	Pt	1.45 ± 0.36	7.73 ± 0.41	12.76 ± 1.31	0.2019 ± 0.0858	3.17	1.441 ± 0.200
Au	SiO_2_	p-Si	–	–	–	–	0.3407 ± 0.0355	36.90	0.900 ± 0.081
Au	NPs/SiO_2_	p-Si	Pt	0.74 ± 0.12	7.08 ± 0.30	3.05 ± 0.02	10.3552 ± 0.3517	1.25	0.059 ± 0.002
Au	Al_2_O_3_/SiO_2_	p-Si	–	–	–	–	0.0005 ± 0.0003	102.21	2485.409 ± 532.769
Au	Al_2_O_3_/NPs/SiO_2_	p-Si	Pt	0.72 ± 0.12	10.92 ± 0.62	4.57 ± 0.06	0.0766 ± 0.0428	22.11	10.741 ± 3.372
Au	Al_2_O_3_/NPs/SiO_2_	p-Si	Pt	0.74 ± 0.12	7.08 ± 0.30	3.05 ± 0.02	0.0206 ± 0.0013	128.18	462.472 ± 78.245
Au	Al_2_O_3_/NPs/SiO_2_	p-Si	Pt	1.11 ± 0.28	5.34 ± 0.48	5.17 ± 0.07	0.6654 ± 0.1264	2.78	5.157 ± 0.577
Au	Al_2_O_3_/NPs/SiO_2_	p-Si	Pt	1.45 ± 0.36	7.73 ± 0.41	12.76 ± 1.31	1.0555 ± 0.0732	0.51	1.543 ± 0.149

**Table 4:** Comparison of the device structure, NP conditions, reverse saturation current, and forward/ reverse current ratio of the metal/Si contact of this work to others.

Ref.	Top Electr-ode	Inter-contact Structures	Semico-nductor	Doping level	Type of NP	NP Diameter	Areal Density	Coverage Rate	Reverse Saturation Current Density	Forward/ Reverse Current Ratio
				/cm^3^		nm	10^12^ /cm^2^	%	(A/cm^2^)	
This work	Ti	Al_2_O_3_	p-Si	1.5 × 10^15^	–	–	–	–	1.0022 ± 0.1940	13.62
This work	Ti	Al_2_O_3_/NPs	p-Si	1.5 × 10^15^	Pt	0.74 ± 0.12	7.08 ± 0.30	3.05 ± 0.02	18.7460 ± 0.5866	1.03
This work	Au	SiO_2_	p-Si	1.5 × 10^15^	–	–	–	–	0.3407 ± 0.0355	36.90
This work	Au	NPs/SiO_2_	p–Si	1.5 × 10^15^	Pt	0.74 ± 0.12	7.08 ± 0.30	3.05 ± 0.02	10.3552 ± 0.3517	1.25
11	W	-	p-Si	1 × 10^19^	–	–	–	–	~2	~1
11	W	NPs	p-Si	1 × 10^19^	Au	~15.6	~0.01		~2	~1.1
11	W	Thin intrinsic p-Si	p-Si	1 × 10^19^	–	–	–	–	~0.2	–
11	W	NPs/ Thin intrinsic p-Si	p-Si	1 × 10^19^	Au	~15.6	~0.01		~1	–
17	Al	–	p-Si	<8 × 10^18^	Au	–	–	–	~0.002	~350
17	Al	NPs	p-Si	<8 × 10^18^	Au	>20	~0.5		~0.002	~500
17	Al	3 Layer NPs	p-Si	<8 × 10^18^	Au	>20	~0.9		~0.004	~1000
17	Al	–	n-Si	<4 × 10^18^	Au	–	–	–	~0.002	~1000
17	Al	NPs	n-Si	<4 × 10^18^	Au	>20	~2.5		~0.03	~66.67
17	Al	3 Layer NPs	n-Si	<4 × 10^18^	Au	>20	~4.3		~0.2	~50
16	Ti	–	p-Ge	1 × 10^15^	–	–	–	–	~20	~1
16	Ti	NPs	p-Ge	1 × 10^15^	Au	10.4 ± 0.7	0.24 ± 0.04		~20	~1
16	Ti	–	n-Ge	1 × 10^17^	–	–	–	–	~0.008	~375
16	Ti	NPs	n-Ge	1 × 10^17^	Au	10.4 ± 0.7	0.24 ± 0.04		~10	~9
