# Supplementary material for: Barrier Modification of Metal-contact on Silicon by Sub-2 nm Platinum Nanoparticles and Thin Dielectrics
Source: Sci Rep. 2016 Apr 28;6:25234. doi: 10.1038/srep25234 (PMC4848504; doi:10.1038/srep25234)
Supplement: Supplementary Information [file srep25234-s1.pdf]

Supplemental Information:

Barrier Modification of Metal-contact on Silicon by Sub-2 nm Platinum Nanoparticles and Thin Dielectrics

Haisheng Zheng [1], Bikram K. Mahajan [1], Sheng C. Su [1], Somik Mukherjee [1], Keshab Gangopadhyay [1,2], Shubhra Gangopadhyay [1, *]

[1] Department of Electrical and Computer Engineering, University of Missouri-Columbia, Missouri 65201, U.S.A.

[2] Nanos Technologies LLC, Columbia, Missouri 65203

* Correspondence and requests for materials should be addressed to S. G. (gangopadhyays@missouri.edu)

I. Regarding the effect of annealing on the NPs shape or diffusion:

We have performed thermal stability test for capped (3 nm Al_2O_3) and uncapped ~1.55 nm size Pt NPs. Annealing was done in a Rapid Thermal Annealing (RTA) chamber with a temperature of 950C sustained for 15 s. The ramp rate was maximized and is around 70 ˚C/s. Based on the TEM study, the temperature dependence of the Pt NP diameter and density for uncapped 30s Pt NPs, and 3 nm Al_2O_3 capped 30s Pt NPs are plot in Figure S 1 (a) and (b). No significant diffusion or coalescence is observed for uncapped Pt NPs with annealing temperature up to 400 ˚C. For capped Pt NPs, they are stable up to 950 ˚C, indicating a successful prevention of the Pt NPs from diffusion and coalescence by the 3 nm Al_2O_3 capping layer. For the Pt NPs capped with the 0.98nm Al_2O_3 used in this study, though it has undergone stability test and it may not provide as good diffusion prevention as the 3 nm one, it is reasonable to expect a higher temperature stability than the uncapped one. Since the annealing of the Cr/Au for the device is performed at 260 ˚C, lower than 400 ˚C, we believe the Pt NPs can survive at this temperature. We also test the contact characteristics before and after annealing. Though we see some improvement in consistency of the contacts, the overall performance is very similar.

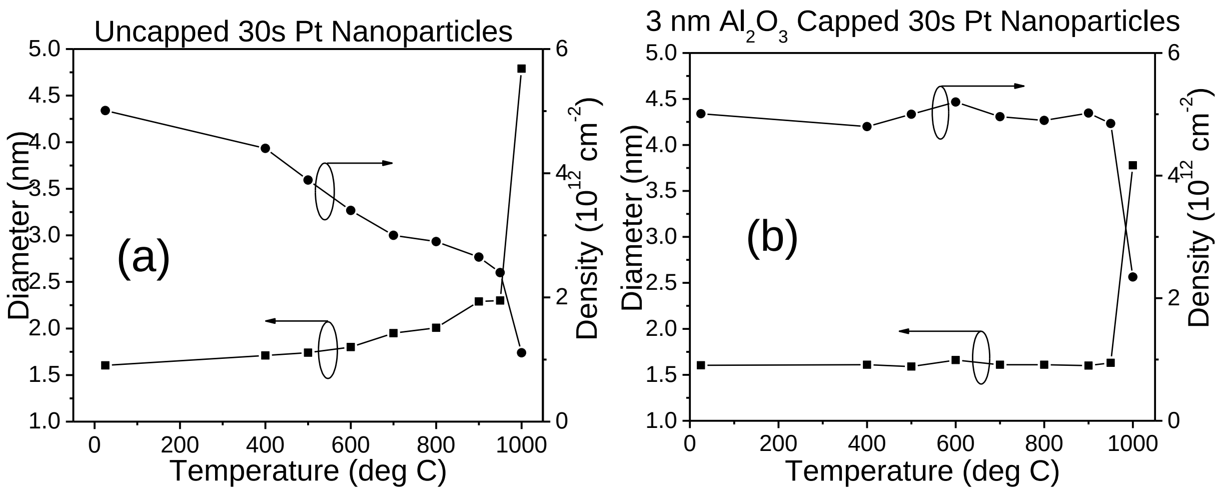

Figure S 1 Comparison of temperature dependence of the Pt NP diameter and density for (a) Uncapped 30s Pt NPs, and (b) 3 nm Al_2O_3 capped 30s Pt NPs

The TEM images of the uncapped particles with various annealing times are plot in Figure S 2.

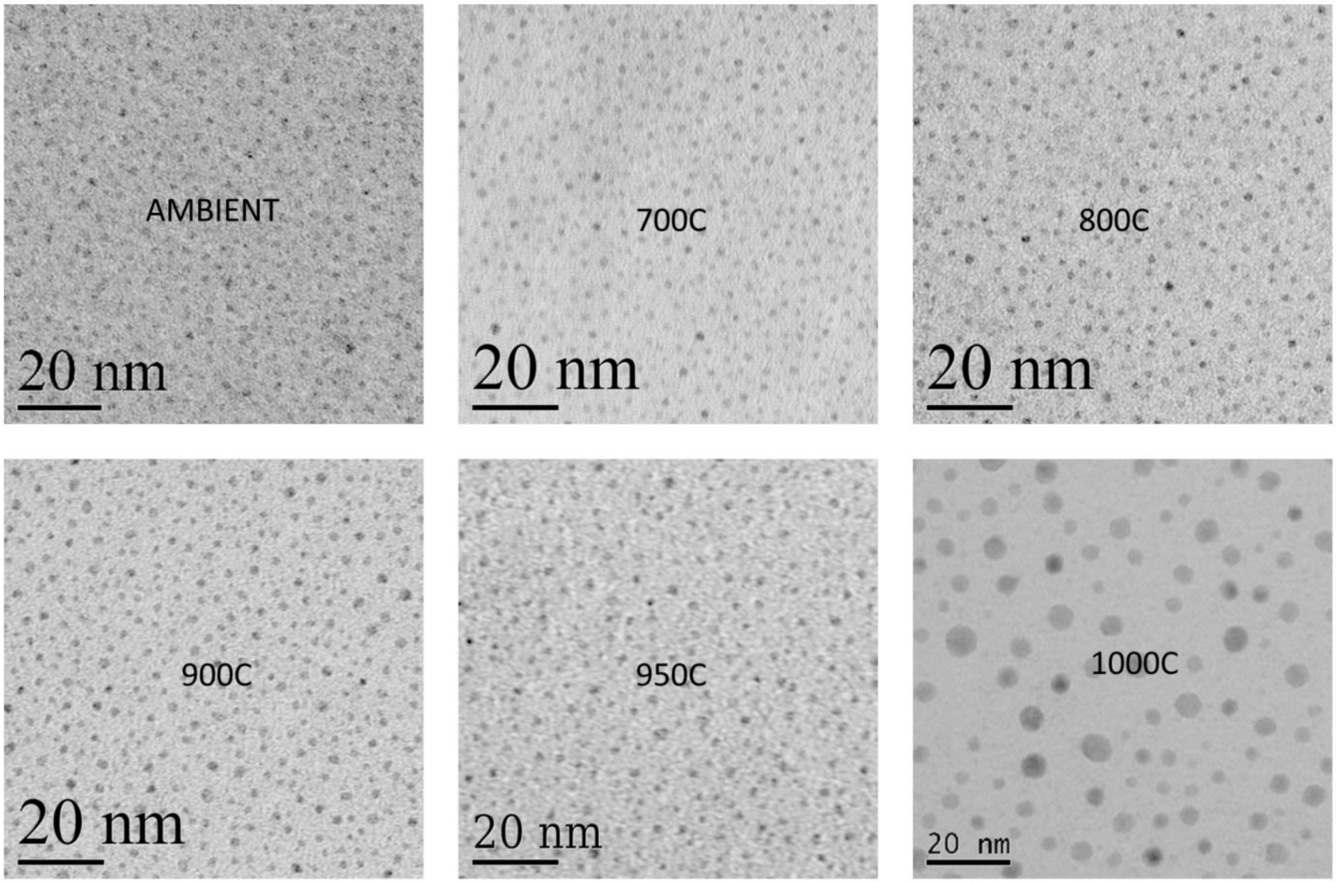

Figure S 2 TEM images of the uncapped particles with various annealing times

The TEM images of the uncapped particles with various annealing times are plot in Figure S 3.

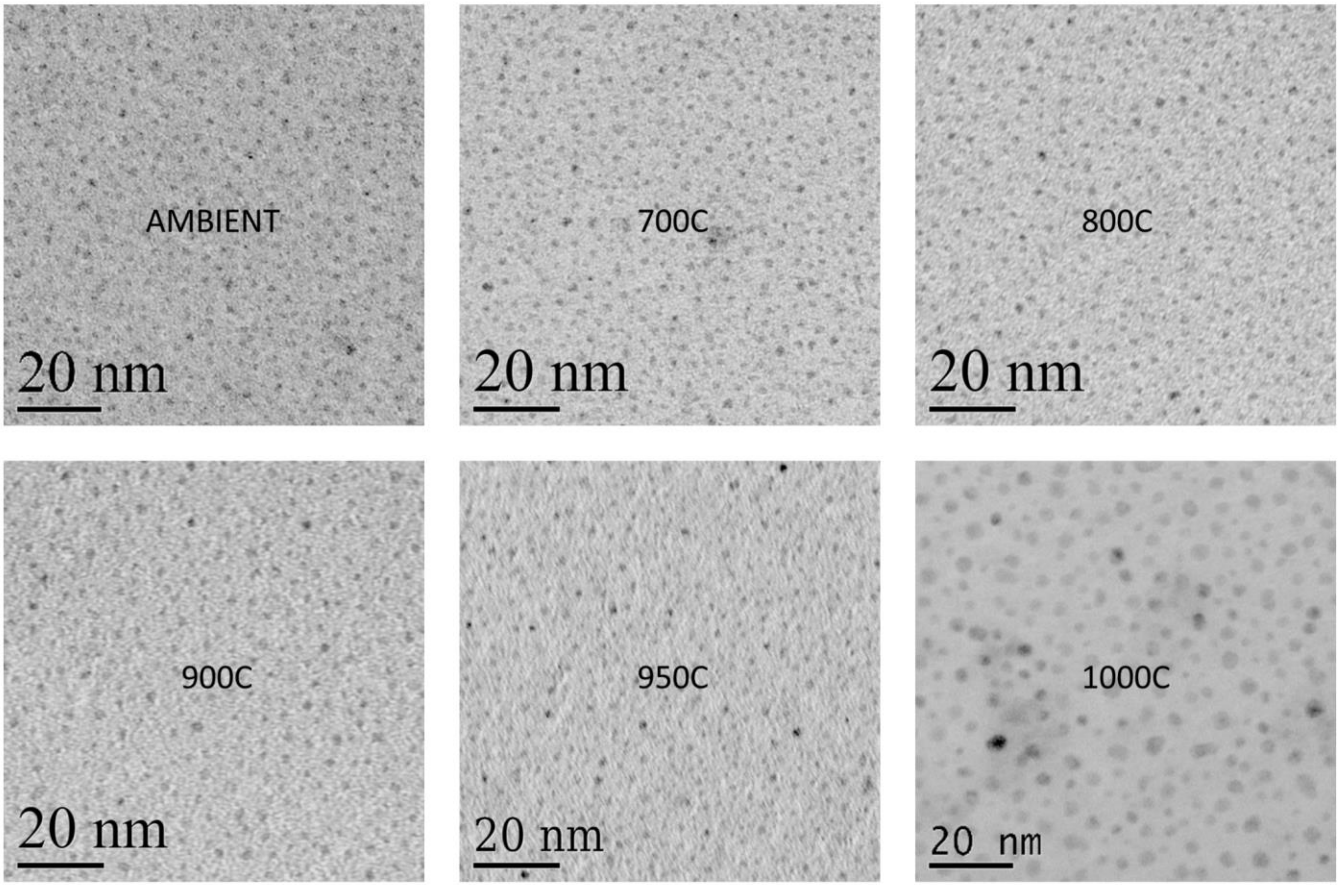

Figure S 3 TEM images of the uncapped particles with various annealing times
